# Deep learning model for identifying significant tricuspid regurgitation using standard 12-lead electrocardiogram

**DOI:** 10.1016/j.ijcrp.2025.200557

**Published:** 2025-12-02

**Authors:** Chun-Chin Chang, Ming-Tsung Hsieh, Yin-Hao Lee, Chih-Hsueh Tseng, Wei-Ming Huang, Ruey-Hsing Chou, Chin-Sheng Lin, Po-Hsun Huang

**Affiliations:** a Cardiovascular Center, Taipei Veterans General Hospital, Taipei, Taiwan; b Cardiovascular Research Center, National Yang Ming Chiao Tung University, Taipei, Taiwan; c Institute of Statistics, National Yang Ming Chiao Tung University, Taiwan; d Division of Cardiology, Department of Medicine, Taipei City Hospital, Yang Ming Branch, Taipei, Taiwan; e Division of Cardiology, Department of Internal Medicine, E-Da Hospital, I-Shou University, Kaohsiung, Taiwan; f Institute of Emergency and Critical Care Medicine, National Yang Ming Chiao Tung University, Taipei, Taiwan; g Department of Internal Medicine, National Yang Ming Chiao Tung University, Taipei, Taiwan; h Department of Critical Care, Taipei Veterans General Hospital, Taipei, Taiwan; i Division of Cardiology, Department of Medicine, Tri-Service General Hospital, Taipei, Taiwan; j Institute of Clinical Medicine, National Yang Ming Chiao Tung University, Taipei, Taiwan

**Keywords:** Tricuspid regurgitation, Artificial intelligence, Deep learning, Electrocardiogram

## Abstract

**Introduction:**

Transthoracic echocardiography (TTE) is the current standard for detecting tricuspid regurgitation (TR); however, it incurs additional costs and is dependent on the operator's skill. In contrast, the 12-lead electrocardiogram (ECG) is widely available during initial evaluations. This study aimed to develop deep learning (DL) models using 12-lead ECG signals and clinical features to detect significant TR.

**Methods:**

Between 2017 and 2019, a total of 5432 patients who underwent both 12-lead ECG and TTE were eligible for this study. Significant TR was identified in 570 of these patients. The DL model architecture was based on a combination of one-dimensional convolutional neural network, efficient channel attention block, and Multihead Attention modules. The model was trained on data from 3910 patients, tested on 435 patients, and validated using both internal and external cohorts.

**Results:**

The diagnostic performance of the DL model using ECG signals, age, and sex to predict significant TR was as follows: an accuracy of 0.762, sensitivity of 0.809, specificity of 0.756, and an area under the curve (AUC) of 0.857. After incorporating additional factors such as RR interval, QRS duration, corrected QT interval, atrial fibrillation, and hypertension into the DL model, the diagnostic performance remained substantial, with an accuracy of 0.762, sensitivity of 0.836, specificity of 0.752, and an AUC of 0.866. External validation of the DL model showed satisfactory results.

**Conclusions:**

Implementing the DL model for ECG interpretation could facilitate the diagnosis of significant TR. However, the clinical utility of this DL model requires further validation and exploration.

## Highlights

• Significant tricuspid regurgitation (TR) is frequently underdiagnosed in routine clinical practice.
• Transthoracic echocardiography is the current gold standard for diagnosing TR, but it is highly operator-dependent.
• The 12-lead electrocardiogram (ECG) is a widely available tool commonly used in the initial screening of cardiovascular diseases.
• Integrating deep learning models into ECG interpretation may offer a valuable approach to aid in the early detection of significant TR.

## Graphical abstract

**Figure:**
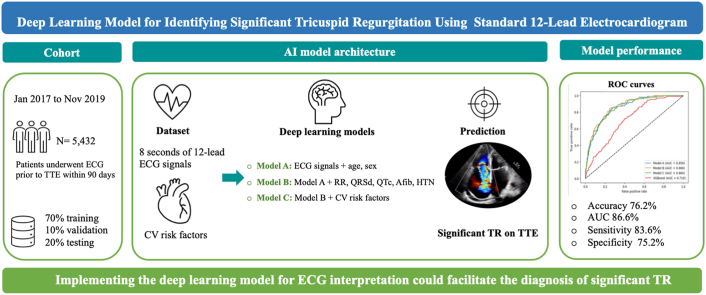

## 1 Introduction

Tricuspid regurgitation (TR) has been forgotten for decades compared with left-side heart valvular disease. Significant TR may be asymptomatic for a long time and therefore is usually underdiagnosed in clinical practice [1]. In addition, significant TR is usually left untreated and has a high surgical mortality rate mainly due to late referral for TR surgery in the past [2].

Transthoracic echocardiography (TTE) is the current standard for evaluation of valvular heart disease (VHD). Nevertheless, TTE needs additional medical costs and is highly operator dependent. The 12-lead electrocardiogram (ECG) is widely available during initial evaluation of cardiovascular disease and can be used easily in screening. ECG criteria suggestive of atrial enlargement or ventricle hypertrophy might be present due to hemodynamic changes caused by underlying conditions, such as arterial or pulmonary hypertension, cardiomyopathy, arrhythmia or VHD. However, there is no individual VHD, irrespective of aortic stenosis, mitral regurgitation, or TR, has specific diagnostic features or validated criteria in ECG.

Nowadays, ECG-based deep learning (DL) models have been investigated in many aspects in cardiovascular disease to identify targeted disease, such as heart failure, coronary artery disease, pulmonary hypertension, or aortic stenosis [[3], [4], [5]]. Significant TR has not yet been tested in DL models.

Recently, transcatheter tricuspid valve intervention is an evolving technology and procedure that is being investigated in many clinical studies [6,7]. Early detection of significant TR using an effective, unexpensive and widely indicated medical device remains an unmet need in clinical practice. We therefore sought to develop DL models bases on 12-lead ECG signals and clinical characteristics that can facilitate the detection of significant TR.

## 2 Methods

### 2.1 Study population

Between 2017 and 2019, a total of 7175 patients who underwent both 12-lead ECG and TTE at Taipei Veterans General Hospital were screened. ECG data within 90 days prior to the TTE was obtained. Repeated patients, patients with pacemakers, and those with heart rate below 40 or above 170 bpm were excluded. After removing the abnormal signal ECGs, a total of 5432 patients remained eligible for this study. Baseline characteristics of these patients were retrospectively collected from medical records. Among the 5432 patients, 570 had significant TR, defined as moderate or severe, while the remaining 4862 patients had no significant TR, categorized as none, trace, or mild. The severity of TR was assessed by independent cardiologists based on TEE.

### 2.2 Electrocardiogram and data pre-process

All ECGs were acquired as digital standard 12-lead ECGs using Philips PageWriter TC70 ECG machine (Philips Healthcare, Andover, MA, USA). ECG raw data were exported from the system to XML files. ECG data were extracted for subsequent preprocessing. To eliminate noise, baseline wander, and powerline interference from raw ECG signals, we employed a bandpass filter with frequencies ranging from 0.05 to 150 Hz, along with a notch filter at 60 Hz to maintain clear biomedical signals. Finally, we chose signals with a length of 8 s to form our dataset. We extracted five morphology features, including RR interval, QRS duration, QT interval, corrected QT interval, and QRS axis, from the filtered ECG data.

### 2.3 Model development

**Fig. 1:**
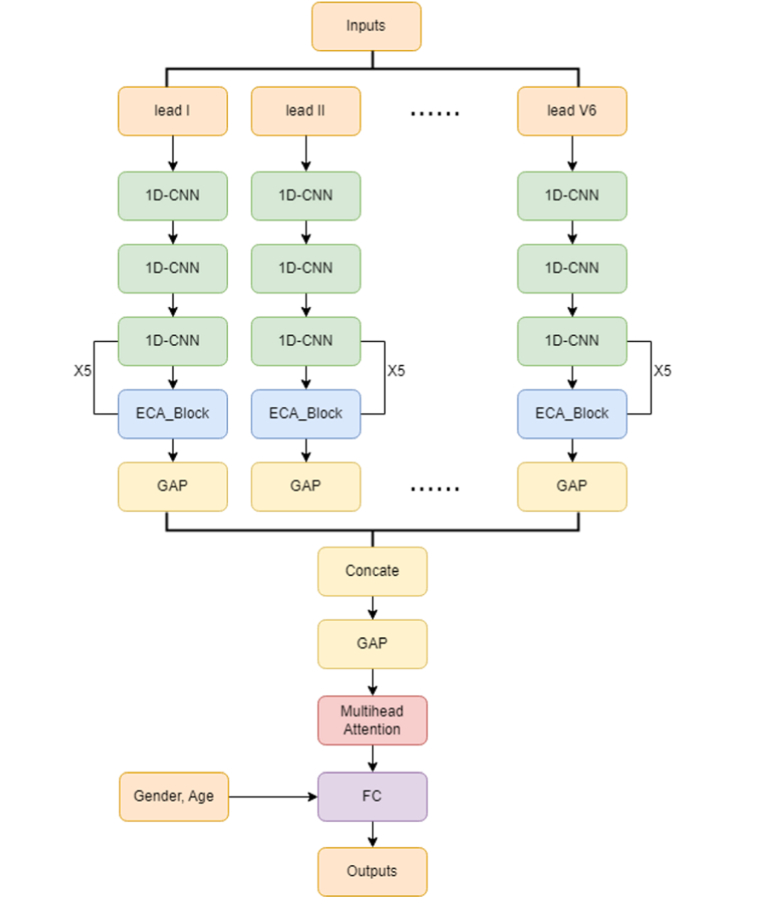
Deep learning model architecture CNN = convolutional neural network; ECA = efficient channel attention.

We constructed a DL model with the architecture depicted in Fig. 1. The model is designed using a combination of one-dimensional convolutional neural network (1D-CNN) for temporal feature extraction, efficient channel attention (ECA) block which adaptively reweights channel-wise features, and Multihead Attention modules that allows the model to focus on multiple aspects of the ECG signal simultaneously and capture long-range dependencies. Various leads of ECG signals were separately input into these modules, and all signal features were concatenated before passing through the fully connected layers. Additionally, we incorporated clinical features such as hypertension, dyslipidaemia, diabetes mellitus, congestive heart failure, chronic kidney disease, chronic obstructive pulmonary disease, previous stroke, previous acute myocardial infarction, and peripheral arterial occlusive disease. Phi coefficients between each clinical variable and significant TR were calculated and Phi coefficient ≥0.1 was used as the predefined threshold for inclusion clinical features in the DL models.

### 2.4 Visualization

**Fig. 2:**
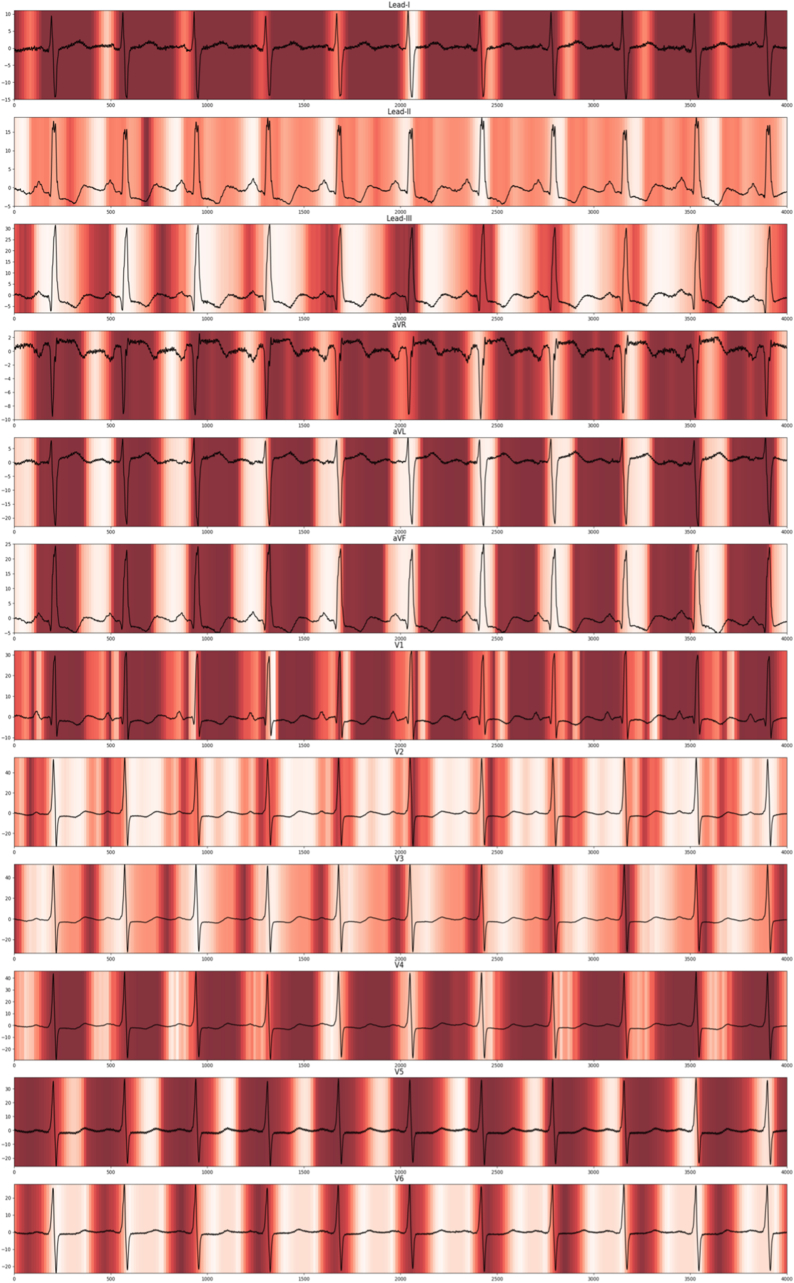
Gradient-weighted Class Activation Map Gradient-weighted class activation map of a representative “true positive” patient with significant TR on TEE.

In this study, Gradient-weighted Class Activation Mapping (Grad-CAM) was used for visualization in our model. Grad-CAM is a technique that visualizes the decision-making process of convolutional neural networks in image classification. By highlighting the important regions in an image that contribute to the model's prediction (Fig. 2), Grad-CAM provides insight into what the model is focusing on. In the resulting visualization, the intensity of colors indicates the importance of different regions. Darker red colors represent areas with higher relevance to the classification decision, while lighter colors indicate regions with less influence. This helps in understanding and interpreting the model's behavior.

### 2.5 Model training

For training the deep learning model, the data was split into a 70 % training set, a 10 % validation set, and a 20 % testing set. The validation set was used to evaluate the model's performance and select the optimal threshold using Youden's J Index. The computational environment for this study included an Intel® Core™ i7-12700K processor and an NVIDIA GeForce RTX 3080 GPU.

### 2.6 External validation

The external validation cohort consisted of ECG data from Tri-Service General Hospital, totalling 2000 patients. After excluding records with abnormal ECG signals, 1785 patients remained for analysis.

### 2.7 Statistical analysis

Model performance metrics including accuracy, sensitivity, specificity, precision, F1 score, and the area under the receiver operating characteristic curve (AUC) were calculated using standard definitions. Confidence intervals were derived using 1000-iteration bootstrapping.

The vital status of the study population was collected to conduct survival analysis. Propensity Score Matching was employed to identify a non-TR group with characteristics like those of the significant TR group. We used logistic regression including 15 covariates (sex, age, RR, QRSd, QTc, hypertension, dyslipidaemia, diabetes mellitus, congestive heart failure, chronic kidney disease, chronic obstructive pulmonary, previous stroke, previous acute myocardial infarction, peripheral arterial obstructive disease and atrial fibrillation) to estimate propensity scores. A 1:1 nearest-neighbor match without replacement was performed, resulting in 587 matched pairs. Standardized mean differences (SMD) were calculated for all covariates. After matching, most covariates demonstrated adequate balance (SMD <0.1). Kaplan–Meier survival curves were generated using follow-up age as the time scale, with mortality as the event outcome. The log-rank test was used for comparison between matched TR and non-TR groups.

## 3 Results

### 3.1 Study population

We used a total of 5432 cleaned ECGs, of which 570 (10.5 %) had significant TR. We randomly selected 3910 patients (72 %) for the training set, 435 patients (8 %) for the validation set, and 1087 patients (20 %) for the testing set. Furthermore, we also additionally collected 2000 ECGs data from Tri-Service General Hospital (TSGH) as an external validation set to evaluate our models. After filtering out abnormal ECGs, there are 1785 ECGs remaining, consisting of 202 TR (11.3 %) and 1583 normal data (Table 1).

**Table 1:** Baseline characteristic of study population.

	Training set (n = 3910)	Validation set (n = 435)	Testing set (n = 1087)	External Validation set (n = 1785)
**Male sex**	2143 (54.8 %)	238 (54.7 %)	548 (50.4 %)	903 (50.6 %)
**Age, years**	62.1 ± 16.8	62.8 ± 17.2	61.6 ± 17.5	62.4 ± 17.4
Age groups				
<40	415 (10.6 %)	43 (9.9 %)	139 (12.8 %)	207 (11.6 %)
40-49	436 (11.2 %)	52 (12.0 %)	106 (9.8 %)	168 (9.4 %)
50-59	713 (18.2 %)	75 (17.2 %)	190 (17.5 %)	348 (19.5 %)
60-69	1019 (26.1 %)	113 (26.0 %)	282 (25.9 %)	419 (23.5 %)
70-79	717 (18.3 %)	66 (15.2 %)	202 (18.6 %)	347 (19.4 %)
≥80	610 (15.6 %)	86 (19.8 %)	168 (15.5 %)	296 (16.6 %)
**Hypertension**	1138 (29.1 %)	118 (27.1 %)	282 (25.9 %)	57 (3.2 %)
**Dyslipidemia**	202 (5.2 %)	23 (5.3 %)	62 (5.7 %)	452 (25.3 %)
**DM**	396 (10.1 %)	34 (7.8 %)	114 (10.5 %)	363 (20.3 %)
**CHF**	82 (2.1 %)	9 (2.1 %)	19 (1.7 %)	111 (6.2 %)
**CKD**	82 (2.1 %)	14 (3.2 %)	19 (1.7 %)	194 (10.9 %)
**COPD**	50 (1.3 %)	4 (0.9 %)	15 (1.4 %)	204 (11.4 %)
**Previous Stroke**	33 (0.8 %)	7 (1.6 %)	4 (0.4 %)	197 (11.0 %)
**Previous AMI**	83 (2.1 %)	12 (2.8 %)	30 (2.8 %)	39 (2.2 %)
**PAOD**	50 (1.3 %)	6 (1.4 %)	5 (0.5 %)	–
**Afib**	354 (9.1 %)	49 (11.3 %)	114 (10.5 %)	67 (3.8 %)
**TR**	408 (10.4 %)	52 (12.0 %)	110 (10.1 %)	202 (11.3 %)

Data was mean ± SD or number (%), Afib = atrial fibrillation; AMI = acute myocardial infarction; CHF = congestive heart failure; CKD = chronic kidney disease; COPD = chronic obstructive pulmonary disease; DM = diabetes mellitus; PAOD = peripheral arterial obstructive disease; TR = tricuspid regurgitation.

### 3.2 Performance of model

**Fig. 3:**
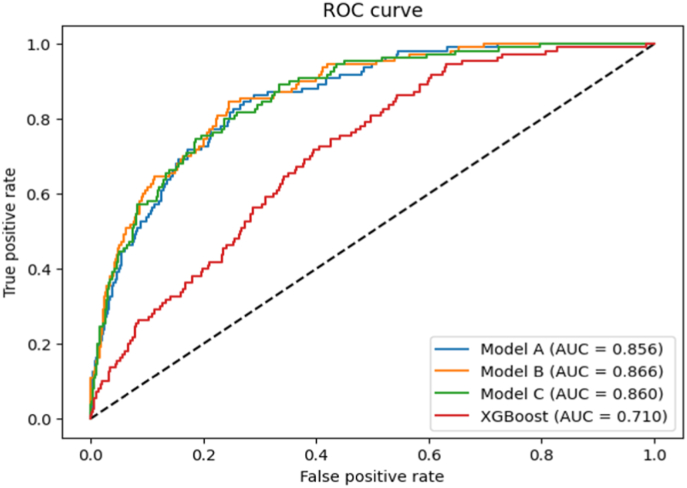
Comparison of receiver operating characteristic curves Model_A: ECG signals + sex, age Model_B: Model_A + RR, QRSd, QTc, Afib, Hypertension Model_C: Model_B + Dyslipidemia, DM, CHF, CKD, COPD, Previous Stroke, Previous AMI, PAOD AUC = area under the receiver operating characteristic curve.

We utilized the ECG signals and clinical factors to generate a correlation coefficient matrix. Based on this matrix, we selected variables with correlation coefficients exceeding 0.1 to train the DL models, including age, RR interval, QRSd, QTc, atrial fibrillation, and hypertension. As shown in Table 2 and Fig. 3, the best model is model B, which included ECG signals along with additional features such as signal features, atrial fibrillation, and hypertension. The diagnostic performance of the model B was substantial, with an accuracy of 0.762, sensitivity of 0.836, specificity of 0.752, and an AUC of 0.866. We conducted the external validation to assess the DL model A. The result showed satisfactory performance: an accuracy of 0.718, sensitivity of 0.644, specificity of 0.728, and an AUC of 0.737.

**Table 2:** Diagnostic performance of deep learning models.

	Internal Validation				External Validation
	Model A	Model B	Model C	XGBoost	
**Accuracy**	0.74 (0.71–0.77)	0.76 (0.74–0.79)	0.77 (0.74–0.79)	0.88 (0.86–0.9)	0.72 (0.69–0.75)
**Sensitivity**	0.84 (0.77–0.9)	0.83 (0.76–0.9)	0.79 (0.7–0.86)	0.12 (0.06–0.18)	0.64 (0.56–0.73)
**Specificity**	0.73 (0.7–0.76)	0.75 (0.72–0.78)	0.76 (0.74–0.79)	0.97 (0.95–0.98)	0.73 (0.7–0.76)
**PPV**	0.26 (0.21–0.3)	0.28 (0.23–0.32)	0.27 (0.22–0.32)	0.28 (0.15–0.41)	0.23 (0.19–0.28)
**NPV**	0.98 (0.96–0.99)	0.98 (0.96–0.99)	0.97 (0.96–0.98)	0.91 (0.89–0.92)	0.94 (0.92–0.96)
**AUC**	0.86 (0.82–0.89)	0.87 (0.83–0.9)	0.86 (0.82–0.89)	0.71 (0.67–0.76)	0.74 (0.68–0.79)
**F1**	0.39 (0.33–0.45)	0.41 (0.36–0.47)	0.41 (0.34–0.46)	0.16 (0.09–0.25)	0.34 (0.28–0.4)
**DOR**	13.78 (8.84–25.12)	15.3 (9.55–28.44)	12.23 (7.51–21.29)	3.74 (1.7–7.3)	4.81 (3.22–7.44)

Model_A: ECG signals + sex, age.

Model_B: Model_A + RR, QRSd, QTc, Afib, Hypertension.

Model_C: Model_B + Dyslipidemia, DM, CHF, CKD, COPD, Previous Stroke, Previous AMI, PAOD.

AUC = area under the receiver operating characteristic curve; DOR = diagnostic odds ratio; NPV = negative predictive value; PPV = positive predictive value.

### 3.3 Survival analysis

**Fig. 4:**
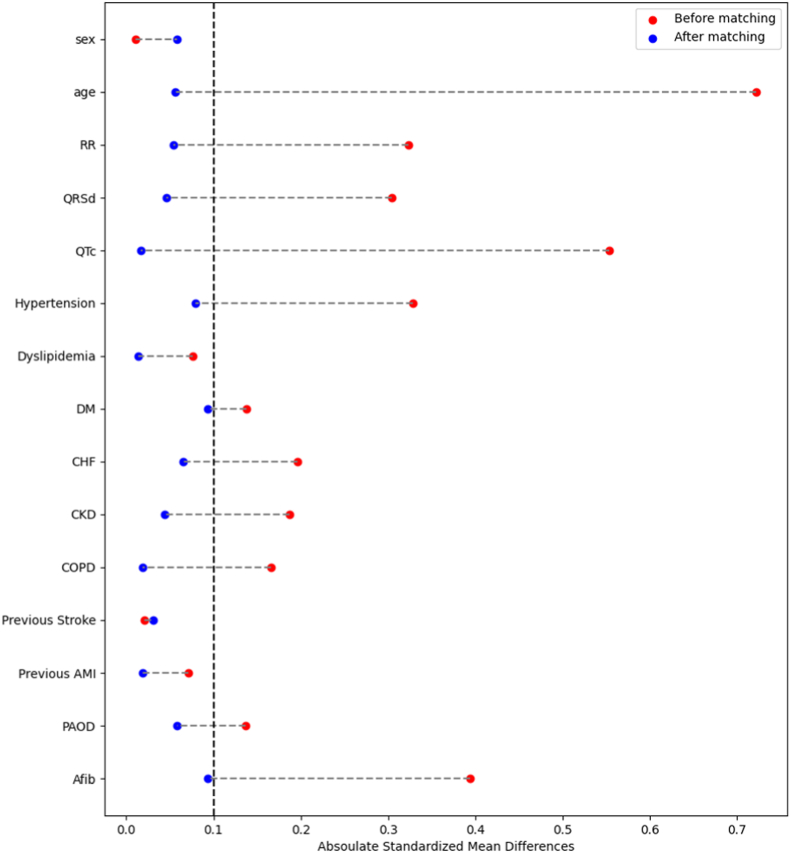
Comparison of data before and after propensity score matching AMI = acute myocardial infarction; Afib = atrial fibrillation, CHF = congestive heart failure; CKD = chronic kidney disease; COPD = chronic obstructive pulmonary disease; PAOD = peripheral arterial obstructive disease.

**Fig. 5:**
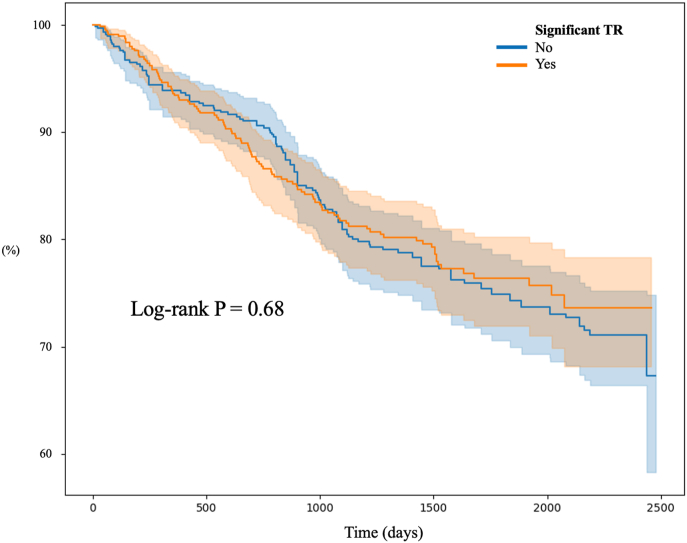
Comparison of Kaplan–Meier survival curves for mortality.

We employed propensity score matching to identify a non-significant TR group with similar characteristics to patients with significant TR (Fig. 4). Subsequently, we utilized the follow-up dates after diagnosis and mortality data to generate Kaplan-Meier curves (Fig. 5). There was no significant difference in lifespan between the significant TR and non-significant TR groups (log-rank P = 0.68).

## 4 Discussion

The present study demonstrated that ECG-based DL models exhibit substantial diagnostic performance in detecting significant TR. Incorporating clinical factors such as atrial fibrillation and hypertension into the DL model can further enhance its performance. To the best of our knowledge, this is the first report on ECG-based DL models for detecting significant TR.

TR is usually asymptomatic for a long period and is difficult to be detected based on physical examination. In contrast to left-side heart valve disease, TR is often underdiagnosed or left untreated in clinical practice, even when the severity of TR is significant. Before the advent of transcatheter tricuspid intervention, patients with symptomatic significant TR were typically treated conservatively rather than undergoing tricuspid valve surgery [8], except in cases of infective endocarditis, combined left-side valve disease, or intracardiac tumors. The reported mortality rate for isolated tricuspid valve surgery was around 8–10 %, primarily due to late diagnosis and referral to surgery [9]. On the other hand, the diagnosis of TR is highly dependent on the TTE, which is not readily available in health-care systems. Patients with significant TR may present with non-cardiac symptoms such as ascites or jaundice, leading them to seek medical consultation from non-cardiologists. In this regard, the early detection of significant TR using an effective, unexpensive and widely available medical device may improve the clinical pathway.

Although ECG is easily available in health-care systems, there is no established criteria to diagnose significant TR based on ECG findings. Significant TR may be accompanied by right atrial enlargement, right ventricular hypertrophy, or right axis deviation on ECG, which are usually observed in patients with elevated pulmonary artery pressure. Recently, Liu et al. reported an ECG-based AI model to identify patients with elevated pulmonary artery pressure [10]. In their cohort, the diagnostic performance of conventional ECG findings for identifying patients with elevated pulmonary artery pressure is relatively low, with sensitivity ranging from 2.4 % to 19.2 %. On the contrary, the ECG-based AI model has an excellent diagnostic performance with sensitivity 81.0 % and specificity 79.6 %. In our study, we did not explore the diagnostic performance of conventional ECG findings for detecting significant TR because there are no established ECG characteristics for diagnosing TR.

Similar to our work, Elias et al. has reported an ECG-based AI model with satisfactory accuracy for predicting left-sided valvular heart disease, including aortic stenosis, aortic regurgitation, and mitral regurgitation [11]. Nevertheless, TR was not included in their study. The positive and negative predictive values were 20 % and 97.6 % in abovementioned study that are in line with the performance of our models. In this context, these ECG-based AI models may serve as a screening modality for early detecting valvular heart disease.

The impact of significant TR on clinical outcomes is well-established. A meta-analysis demonstrated that significant TR is associated with a two-fold increased risk of all-cause death compared with no or mild TR [12]. Topilsky et al. reported that quantitative measures of TR were linked to increased mortality in patients with heart failure. These evidences may support earlier diagnosis and intervention for significant TR [13]. In our study, patients with significant TR had a similar mortality risk compared to a propensity score-matched cohort without TR. This finding may be attributed to unadjusted confounding factors and the inherent limitations of a retrospective study. Moreover, other competing causes of mortality might attenuate the independent effect of TR on survival outcomes in this clinical population.

There are several limitations in our study. First, the study population was selected from a single center and underwent 12-lead ECG and TTE, suggesting they may have a higher likelihood of having cardiovascular disease compared to the general population. Second, the TR severity was reported by echocardiography cardiologists from routine clinical practice rather than by a central core laboratory. Lastly, the diagnostic accuracy of our model was slightly decreased in the external validation cohort, highlighting the need for federated learning and validation.

In conclusion, implementing the DL model for ECG interpretation could facilitate the diagnosis of significant TR. This ECG-based DL model may serve as a screening modality in clinical practice.

## CRediT authorship contribution statement

**Chun-Chin Chang:** Writing – original draft, Project administration, Data curation, Conceptualization. **Ming-Tsung Hsieh:** Writing – original draft, Software, Methodology, Formal analysis, Data curation. **Yin-Hao Lee:** Writing – review & editing, Methodology, Data curation. **Chih-Hsueh Tseng:** Writing – review & editing, Methodology, Data curation. **Wei-Ming Huang:** Writing – review & editing, Methodology, Data curation. **Ruey-Hsing Chou:** Writing – review & editing, Resources, Data curation. **Chin-Sheng Lin:** Writing – review & editing, Formal analysis, Data curation. **Po-Hsun Huang:** Writing – review & editing, Supervision, Project administration, Conceptualization.

## Ethical statement

The study was approved by the research ethics committee of Taipei Veterans General Hospital and was conducted following the Declaration of Helsinki.

## Data availability statement

The medical records dataset used in this study is not publicly available due to the need to protect patient privacy.
